# Granting Leave to Patients in Bavarian Forensic-Psychiatric Hospitals: A Survey to Describe the Current Process and Develop Guidelines

**DOI:** 10.3389/fpsyt.2020.00287

**Published:** 2020-04-15

**Authors:** Halina Sklenarova, Janina Neutze, Thomas Kretschmer, Joachim Nitschke

**Affiliations:** Forensic Psychiatric Clinic, Ansbach District Hospital, Ansbach, Germany

**Keywords:** forensic psychiatry, forensic patients, standardized intervention, risk assessment, reoffending

## Abstract

Forensic-psychiatric patients reoffending or absconding during the leave granted to them (hereafter referred to as “granted leave”) have gained increased attention by researchers and the general public. The patients’ right to freedom on the one hand and the need for protection of the general public from serious harm on the other hand represent broadly discussed ethical issues. Thus, demands on quality regarding decisions on patients’ granted leaves might be high. Despite such requirements, research on decision-making processes regarding granting leave in forensic psychiatry is very limited and focuses primarily on particular aspects. The present study aims at providing a first overview of the decision-making processes regarding granted leave in forensic psychiatry as a whole. Furthermore, the link between the particular steps of the process and absconding should be explored. In this way, the study results should contribute to provide a theoretical framework for the development of guidelines concerning granted leave in forensic psychiatry. A combination of qualitative and quantitative approaches will be used to collect data: information about risk assessment, decisions on granted leave, and documentation systems in forensic psychiatry will be collected *via* semi-structured interviews and quantified for further analyses using a checklist developed for this study; data on the implementation of risk assessment tools and documented patient information will be obtained *via* two self-constructed questionnaires; information about the absolute number of abscondences per hospital will be obtained from the Bavarian Authority for Forensic Commitment. The sample will include staff from all 13 forensic-psychiatric hospitals in Bavaria (Germany) comprising six professional groups: hospital directors, security officers, complementary therapists, psychiatrists, psychologists, social workers, and nursing staff. In each hospital, at least one member of each professional group should participate in the study. In total, 151 interviews will be held. As the study goals are descriptive, there are no pre-formulated hypotheses. Developing guidelines would be the first step towards further standardization of the granted leave decisional process in forensic psychiatry and to make it more transparent for patients, staff members, hospital directors, and the government.

## Introduction

Recent incidents such as reoffending by forensic-psychiatric patients on leave granted to them (hereafter referred to as “granted leave,”) in Germany have raised several key questions on the quality and comprehensibility of decision-making regarding the granted leave and discharge process. Neumann and colleagues (1) reported absconding rates from forensic-psychiatric units in the Land of Lower Saxony (a federal state in Germany) between 2000 and 2015 to be under 10%, delinquent acts were in the per-thousand range. According to the literature review from Campagnolo and colleagues (2), absconding rates from forensic-psychiatric units in predominantly English-speaking countries reach from “rare” up to 20%, violent acts were reported to be “infrequent”. However, many published studies do not quantify absconding rates, and the definitions of abscondence and studied populations differ substantially (2).

The core definition of forensic psychiatry covers the assessment and treatment of individuals with mental disorder, who show antisocial or violent behavior (3). It is commonly assumed that a mental disorder impairs cognition, perception, emotion, and judgment of an individual. This might result in full or partial insanity defense of an offender. According to the German Criminal Code (GCC, 4), individuals committing offenses are not or only partially responsible for their behavior if they suffered from a mental illness at the time of the offense. Moreover, the mental disorder must substantially affect their insight and control capabilities (Sections 20,21; GCC; 4). If an offender with a full or partial insanity defense is assumed to be a substantial threat to the general public, a detention in a forensic-psychiatric hospital can be ordered instead of or complementary to imprisonment (Section 63; GCC; 4, see also 5). Additionally, there is a specific legal provision for offenses related to substance abuse in Germany (6, 7).

The legal frameworks governing the detention and treatment in forensic psychiatry vary across European countries and even across Germany (5, 8). The similarity of forensic-psychiatric care concerns the fact that offenders with an insanity defense are treated until they show sufficient therapeutic change and reduced risk for reoffending. Thus, the treatment is not time-limited, but lasts until the patients’ recovery in order to protect the general public from further serious harm (9). This has, however, raised several ethical issues (10, 11). One major concern refers to the patients’ right to freedom (12). Considering this concern, German forensic-psychiatric hospitals are required to minimize the length of patients’ stay by, among others, offering granted leave (13). Such procedure shall encourage patients’ rehabilitation and motivate them to participate in further treatment (14, 15). In Bavaria, the clinical practice in forensic psychiatry consists of granting leaves to patients, which are temporally limited and gradually escorted, unescorted, inside and offside a high security area (16).

Each decision to grant one of these leaves requires a careful assessment of the risk of reoffending, substance misuse or absconding, and comprises a crucial decision process (17). However, research on how granted leave decisions are made and implemented is limited. While considerable research attention has been directed towards violence and recidivism prediction after discharge (18–22), little attention has been paid to the prediction of in-hospital violence (23) or absconding of forensic-psychiatric patients (24, 25). Moreover, studies on granted leave decisions have looked primarily on particular aspects such as clinical decision-making, clinicians’ subjective perspectives, or novel interactions (14, 26, 27). With respect to risk assessment Simpson and colleagues (28) found evidence for lower absconding rates after implementation of structured professional judgment (SPJ, 29) within an interdisciplinary team in the decision-making process of granted leave.

In summary, many questions regarding granted leave decisions remain unanswered; for example, whether decisions are guided by structured judgments such as SPJ (29) or how clinicians come to a decision and which particular aspects are considered. Existing studies have been conducted in English-speaking countries or the Netherlands (14, 26). So far, data for German-speaking countries have been collected in just one study (1).

### How This Study Will Contribute to the Field of Research and Literature

The topic of granted leave deserves more scholarly attention, especially because of its impact on the general public. To the authors’ best knowledge, no study has looked specifically into the granted leave decision process as a whole and the extent of its standardization in Germany. Thus, this study will be conducted to investigate this process and understand its nature better. The aim of this study is two-fold. First, it should provide an overview about the current state of the granted leave decision processes at all forensic-psychiatric hospitals in Bavaria (Germany). In the process, differences and similarities between these 13 hospitals will be addressed. Second, a link between the process-steps and the number and quality of abscondences from granted leave should be identified. Based on this information recommendations for guidelines concerning the target process will be developed to progress towards the standardization of the decision-making process concerning granted leaves in forensic-psychiatric hospitals in Germany.

## Methods and Analyses

The present study will implement a combination of qualitative and quantitative approaches. The qualitative data will be collected using semi-structured interviews (conducted with forensic-psychiatric hospital staff) and subsequently transformed into quantitative data. Additional quantitative data will be obtained *via* two questionnaires designed for the purpose of this study. The data will be analyzed primarily quantitatively. The qualitative data should (1) guarantee to obtain as much information as possible and (2) be supportive in understanding the hospital policies. The rates of abscondences during granted leave at hospital level will be obtained from the Authority for Forensic Commitment in Bavaria.

### Study Design

The study will not be a clinical trial. It is designed as a descriptive, cross-sectional study with mixed-method data collection.

### Selection of Subjects

The study sample will include representatives of the forensic-psychiatric hospital comprising six professional groups: psychiatrists, psychologists, social workers, complementary therapists, security officers, and nursing staff.

Study participants will be selected from all 13 Bavarian forensic-psychiatric hospitals of which the granted leave is a part of the therapeutic intervention. Participants will be recruited using a nonprobability purposive sampling procedure (30). First, the director of each hospital will be recruited *via* study investigators. Afterwards, the directors will recruit further employees in their hospitals, fulfilling the inclusion criteria for study participation: a work experience at the particular forensic-psychiatric hospital of at least 1 year and the affiliation to one of the professional groups mentioned above.

In 12 participating forensic-psychiatric hospitals, 12 staff members will be interviewed respectively: the hospital director/head of the forensic psychiatric department, one security officer, two complementary therapists, two psychiatrists, two psychologists, two social workers, and two members of the nursing staff (one for each field of disorders—addiction vs. other psychiatric diagnosis). In one forensic-psychiatric hospital, only patients with substance misuse and/or addiction are treated, thus besides psychiatric chief, only one psychiatrist, one psychologist, one social worker, one occupational therapist, one security officer, and one member of the nursing staff will be interviewed. Therefore, a total of 151 interviews will be held.

### Data Collection

The participants will be asked to participate in a semi-structured interview, which would take 1–1.5 h on average. All interviews will be conducted face-to-face at each of the 13 Bavarian forensic-psychiatric hospitals in situ. The interviews will not be audiotaped, but the interviewers will create transcripts from their interview notes immediately after the interview. Subsequently, qualitative data will be quantified using items of the checklist developed for this purpose (see *Measures and Outcomes*) to conduct inferential statistics (31). The checklist will be completed after each interview by a study investigator other than the interviewer for data protection reasons.

Additionally, all participants will need to complete two questionnaires seeking details of (1) the documented patient information and (2) the use of risk assessment tools. Completing the two questionnaires would approximately require half an hour; thus, the time investment for the entire study participation shall not exceed 2 h. Supplementary data will be gathered from randomly selected patients’ files in each participating forensic-psychiatric hospital to verify the reported information. With the planned sampling procedure, we seek to achieve a full participation.

### Measures and Outcomes

We designed four instruments to provide detailed information on the granted leave decision-making in Bavarian forensic-psychiatric hospitals.

#### Checklist

Following a deductive approach to analyze qualitative data (32), a checklist was designed according to the guidelines to develop checklists (33). The checklist enables the transformation of qualitative interviews into quantitative data to obtain information on the degree of implementation of the apriori defined relevant procedural steps in granted leave decisions in the participating hospitals.

**Figure 1:**
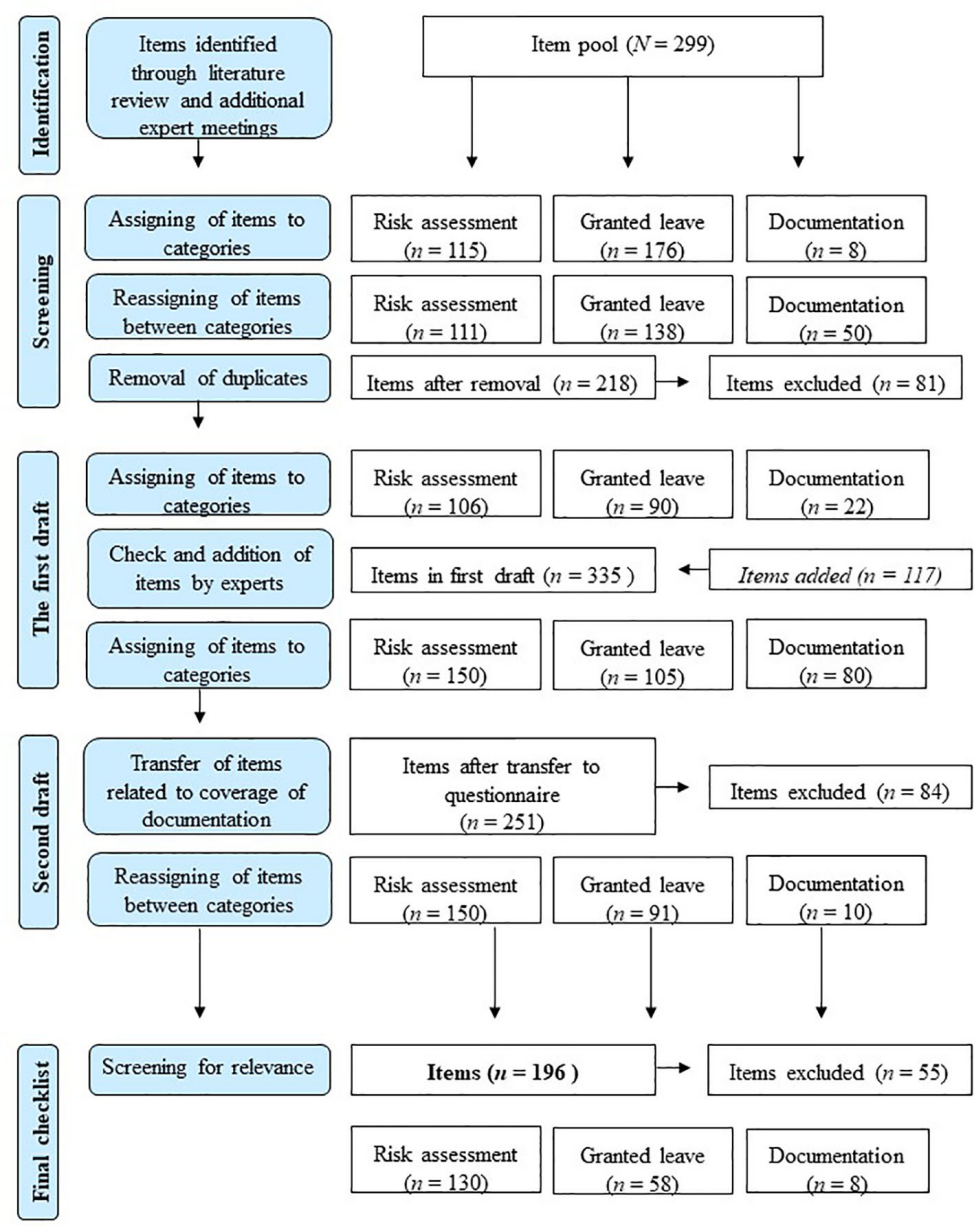
Development of the checklist.

**Figure 1** illustrates three developmental phases of the final checklist. The final checklist consists of 196 items (particular process steps) subdivided into three assessment categories: risk assessment based on SPJ (130 items), granted leave process required by the legislation and recommended by experts (58 Items) and description of documentation systems (8 Items). The items were selected based on a review of the relevant literature with respect to published expert recommendations (34–36), Bavarian legal requirements for granted leave (16), and SPJ as an international standard (29, 37–43). Additional items were added after expert meetings (psychologists and psychiatrists). In total, 167 items are rated on a 4-point scale, and the response options vary depending on the item. For example: *Multiprofessional decision at each granted leave level* (*0—process section not available, 1—process section partially available, 2—process section available but not standardized, 3—process section fully available and standardized).* In total, 29 items are rated dichotomously (*0—no, 1—yes*). Based on the rating, an overall sum score can be built ranging from 0 to 530. High scores represent high implementation of empirical-based and by law required granted leave decision process steps. In order to provide more differentiated information, the overall sum score can be divided into three subscale scores for risk assessment based on SPJ, granted leave based on experts recommendation and Bavarian legal requirements, and documentation systems. Some items will be weighted differently for further statistical analyses. The checklist does not represent a validated measurement, but the interrater reliability will be determined after completing the pilot study. Wertz and colleagues (44) conducted research on the quality of forensic-psychiatric expert reports using a similar method, achieving a substantial interrater reliability (r =.78).

#### Semi-Structured Interview

The semi-structured interview was developed in order to gain the information needed to rate the checklist (see **Figure 1**). The interview questions (in total 53) are of two kinds: 17 open questions addressing the three main assessment categories (risk assessment, granted leave, and documentation systems) and 36 subsequent optional questions to prevent missing data in the checklist. Additionally, each participant will be asked for a personal rating of the standardization of the decisional process regarding granted leave *via* six items rated on a 4-point scale (two for each topic). For example: *How standardized do you perceive the granted leave process at your hospital to be? (1—not standardized, 2—rather not standardized, 3—rather standardized, 4—fully standardized)*.

#### Documentation Questionnaire

To obtain further information on the hospitals mode of recording patients’ medical and forensic information we decided to develop a questionnaire in addition to the checklist and the interview (see **Figure 1**). While the interview provides information on the medical documentation systems, the questionnaire aims an economic assessment of the coverage of recorded clinical information. It consists of 72 self-rating questions including items regarding records of general patient information (e.g. offense, diagnosis and therapy), granted leave (e.g. granted leave level) and risk relevant information (e.g. absconding or ratings from risk assessment tools). The items were chosen based on the literature review (SPJ and legal requirements) and expert meetings (psychologists and psychiatrists). Each item is rated on a 4-point scale. For example: *How often is the granted leave level documented in patients’ reports?* (*0—never, 1—infrequent, 2—often, 3—always)*. For each item, it is possible to state “*I do not know*.” As some hospitals might store parts of the patients’ information both digitally (and thus generally accessible) and on paper, the items have to be rated on two levels: documentation on the ward and hospital level. For example, *the current diagnosis (International Classification of Diseases-10)* (45) *is documented 3—always* on the ward level and *2—often* on the hospital level. An overall sum score can range from 0 to 216 for each level. Additionally, three subscale scores can be identified (for each level) ranging from 0 to 111, 48, and 57 for documentation of patient information, granted leave information, and risk relevant information respectively. Higher scores indicate higher amounts of documented information. The questionnaire shall provide solely descriptive information and is not a validated instrument.

#### Use of Risk Assessment Tools Questionnaire

Structured, empirically derived, and theoretically driven risk assessment tools should play a central role in decision-making pertaining to sentencing, release, case management, and the selection of rehabilitation methods (46), as they may contribute to the prediction of recidivism in violent and sexual offenders (18, 20).

**Figure 2:**
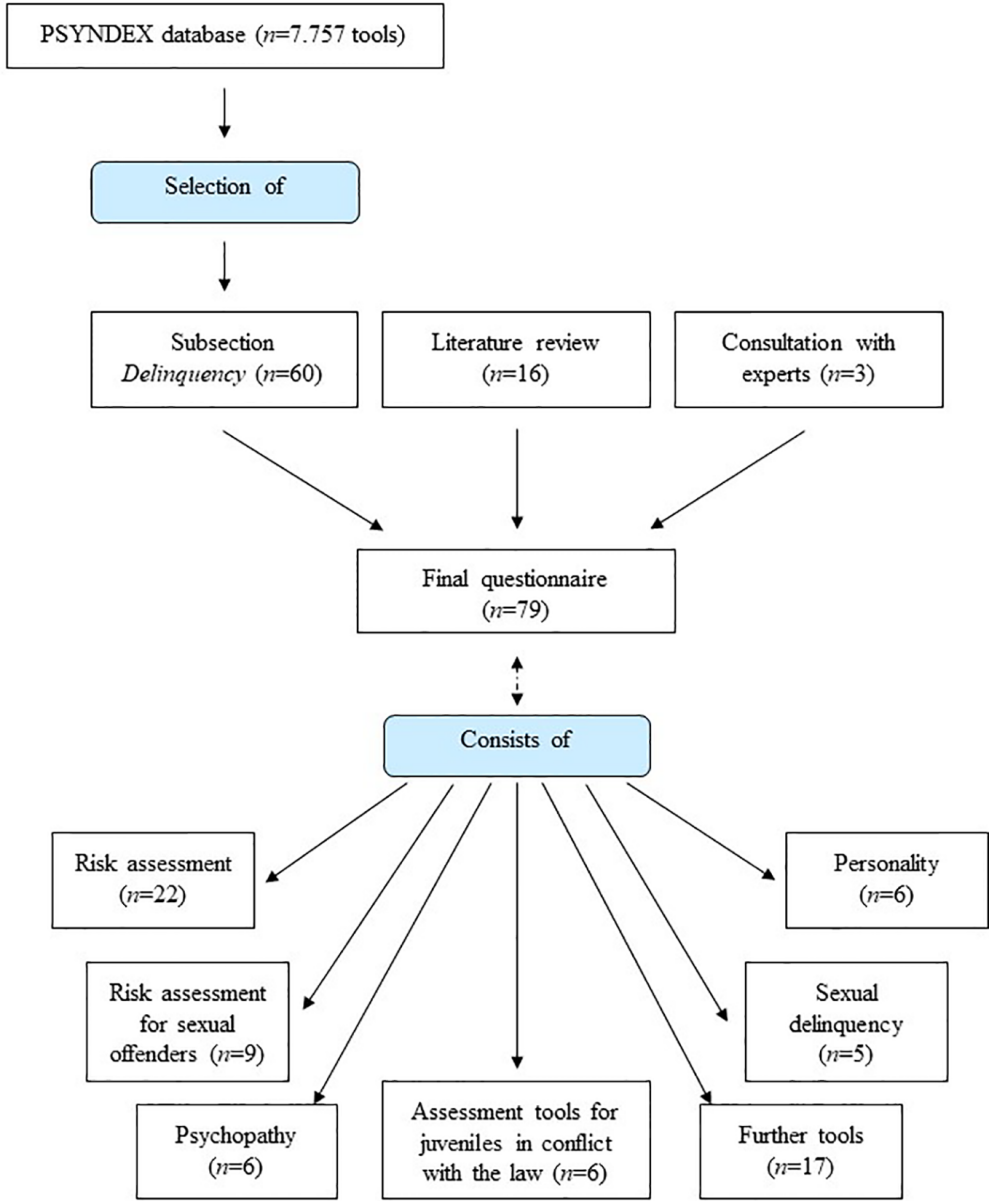
Questionnaire on risk assessment tools—development process.

The implementation of risk assessment tools in the process of decisions regarding granted leave within the Bavarian forensic-psychiatric hospitals involved will be measured *via* a questionnaire listing tools used in German-speaking countries. The final version of the questionnaire includes 79 tools (for selection process: see **Figure 2**) and is a compromise between an extensive list (on the one hand with the risk of requiring too much time and cognitive investment by the participants on that specific issue, on the other hand with the advantage that sporadically used tools can be recognized) and a short list (with the risk that the interviewee omits/forgets infrequently used tools).

The list of tools was prepared based on the PSYNDEX database (run by The Leibniz Institute for Psychology Information (47), a supra-regional scientific research support organization for psychology in German-speaking countries) that contains descriptions of tests, rating scales, questionnaires, observation methods, and other diagnostic instruments used in German-speaking countries. Section 090800 on “tests measuring the tendency for delinquent behaviour/attitudes” (48) contains 60 tools. In addition, tools have been added on the basis of literature review (23, 41, 49; 16 tools added) and after the consultation with experts (three tools). The tools have been clustered into sub-groups called “risk assessment” (22 tools), “risk assessment for sexual offenders” (9 tools), “psychopathy” (six tools), “personality” (six tools), “delinquency” (three tools), “sexual delinquency” (five tools), “further tools” (17 tools), and “assessment tools for juveniles in conflict with the law” (11 tools). At the end of the questionnaire, interviewees could add and include tools that have not been listed before.

It has to be emphasized that the selection process of the list of tools aims to obtain a detailed picture of the tools uses (which might imply the presence of tools that are not validated and without standard values, or the use of tools for other purposes than what it was for what it was developed); it is by no means an evaluation of the quality of the tools nor a definition of the intended purpose of the tool. In the questionnaire, the frequency of use for each tool has to be estimated on a 4-point rating scale labeled *0—*”*never,*” *1—*”*infrequent,*” *2—*”*often,*” and *3—*”*always*”; estimation can be omitted if the tool or its use within the hospital is unknown to the interviewee.

### Data Management

By using mixed methods for collecting qualitative and quantitative data, we aim to obtain high-quality data, as the qualitative data enable us to gather more extensive information than only questionnaires (50).

#### Data Access

The interview transcripts and data from the questionnaires and checklists will be stored using pseudonyms and digitally in a collaborative file on a hospital server to protect them from accidental loss or improper access. Only study members can access the digital data.

#### Data Security

To ensure that none of the collected data can be associated with a certain study participant nor a particular forensic-psychiatric hospital, we will undertake a two-step pseudonymization. 1) Based on a code-list created for the purpose of the study, an individual code will be assigned to each participating hospital. This code-list will be known to only one person, who is not a study member. After the collection of all data, the code-list will be destroyed. 2) To enable the withdrawal at any moment, each participant will receive a self-created pseudonym. This will consist of the first two letters of his/her father’s first name, the first two letters of his/her mother’s first name, and his/her mother’s birth month as a number. The data will be stored as Microsoft Excel files, and all statistical analyses will be conducted using R-Statistics (51).

### Anticipated Results and Data Analyses

For the purpose of the study, we will not attempt to prove pre-formulated hypotheses, but want to provide detailed information about the decisional process of granted leave in forensic-psychiatric hospitals in Bavaria, Germany. Therefore, descriptive statistics will be calculated predominantly. With respect to differences and similarities between the participating forensic-psychiatric hospitals, and to estimate the relationship between the process steps and abscondences, inferential statistics based on the general linear model and comparisons of means and frequencies will be calculated. The data will be analyzed on hospital and field of treatment (addiction vs. other mental disorders) level.

One question of the study addresses the agreement between the hospital directors and the staff members regarding the described decisional process of granted leave. To determine this, interrater reliability between the hospital director and staff members will be calculated. To further validate the stated frequency of use of risk assessment tools and the amount of documented patient information, a total of 10 patients’ records will be randomly and anonymously selected at each hospital to be compared to the gathered interview data. After the pilot study, the interrater reliability for the developed checklist will be calculated and all measurements will be adjusted.

The development of the anticipated guidelines based on the study results will follow the reporting tool for practice guidelines in healthcare: the RIGHT statement (52).

### Potential Limitations

Although the study is planned carefully, there are several limitations and potential pitfalls. With the chosen sampling procedure, the risk of participation bias cannot be prevented or minimized. Thus, our result might be biased by the non-random sample. This, however, should not substantially affect the quality of the results regarding our research questions. Furthermore, the social desirability bias should be presumed and discussed critically while presenting or publishing the study results. It cannot be ruled out, that participants will try to portray their hospitals favorably, though data will be pseudonymized such that conclusions cannot be drawn regarding the 13 hospitals or individual participants. With respect to the potential interviewer bias, we tried to avoid this using standardized interview questions and by training the interviewers before and during the pilot study. Finally, the data collection and targeted full participation entail a high level of difficulty. As the interview duration amounts up to 2 h and the potential study participants would face a lot of unexpected incidents (e.g., phone calls), an extraordinary time management is indispensable during the data collection to achieve the study goals and maintain the time schedule.

## Discussion

This study is undertaken to make the first steps in scholarly research in the field of decisions about granting leave in German forensic-psychiatric hospitals. Our findings could have several practical and scientific implications. First, the descriptive results and following recommendations would make the decisional process more transparent for staff members and governmental authorities. Moreover, with a higher level of standardization, young professionals can be trained faster, which will save time and economical resources. Further, the granted leave decisional process will become more transparent for patients, especially with respect to stable requirements they have to fulfill in order to obtain granted leaves. Lastly, more standardization should avoid the risk that patients granted leave would reoffend or abscond and thus, protect the general public against potential harm.

Beside these practical implications, our data shall also provide preliminary evidence and theoretical support for further research. As our findings will not be generalizable to other federal states of Germany, we want to encourage researchers to examine this in the other German federal states to obtain more empirical support for the development guidelines across federal states. More standardization should also help researchers to compare their data better, for example with respect to risk assessment of the patients on different levels of granted leave. It is important to note, that the development of recommendations shall provide the first step towards more standardization in forensic-psychiatric settings. However, more research will be necessary to evaluate the recommendations and to gain more empirical data.

## Study Status

The study duration is set for 48 months. Presently, a pilot study has been initiated in June 2019 in two forensic-psychiatric hospitals to verify the interrater reliability of the developed checklist and train the interviewers. The other 11 forensic-psychiatric hospitals in Bavaria received a written request for recruitment for the study participation in August 2019.

## Ethics Statement

The studies involving human participants were reviewed and approved by the German Psychological Sociey (Deutsche Gesellschaft für Psychologie, DGPs), Approval Number: 2019-10-18VA, Date of approval 2019-11-12. The patients/participants provided their written informed consent to participate in this study. Written informed consent was obtained from the individual(s) for the publication of any potentially identifiable images or data included in this article.

## Author Contributions

JNi, HS, JNe, and TK designed the study. HS, JNe, and TK will collect the data. HS drafted the manuscript assisted by JNe. JNi, as principal investigator, drafted the study proposal to receive grants and carried out the crucial revision of this manuscript. All authors read, edited, and approved the final manuscript.

## Funding

The study received a financial support from the Bavarian Centre for Families and Social Affairs (ZBFS).

## Conflict of Interest

The authors declare that the research was conducted in the absence of any commercial or financial relationships that could be construed as a potential conflict of interest.

## References

[1] Neumann M Heintzsch R Glaubitz C Killig L Schumann R Bliesener T Analyse der Vollzugslockerungen im niedersächsischen Maßregelvollzug Hannover: Criminological Research Institute of Lower Saxony (KFN) (2019). https://www.researchgate.net/profile/Merten_Neumann/publication/336305218_Analyse_der_Vollzugslockerungen_im_niedersachsischen_Massregelvollzug/links/5d9af8aea6fdccfd0e7f3315/Analyse-der-Vollzugslockerungen-im-niedersaechsischen-Massregelvollzug.pdf (Accessed January 27, 2020).

[2] Campagnolo D Furimsky I Chaimowitz G Absconsion from forensic psychiatric institutions: a review of the literature. Int J Risk Recovery (2019) 2(2):36–50. 10.15173/ijrr.v2i2.3920

[3] Gordon H Lindqvist P Forensic psychiatry in Europe. Psychiatr Bull (2007) 31(11):421–4. 10.1192/pb.bp.107.014803

[4] Fischer T Schwarz O Dreher E Tröndle H (2014). Strafgesetzbuch: StGB.

[5] Müller-Isberner R Freese R Jöckel D Gonzalez CS Forensic psychiatric assessment and treatment in Germany. Legal framework, recent developments, and current practice. Int J Law Psychiatry (2000) 23(5-6):467. 10.1016/s0160-2527(00)00056-x 11143945

[6] Nedopil N Ottermann B Treatment of mentally ill offenders in Germany: With special reference to the newest forensic hospital—Straubing in Bavaria. Int J Law Psychiatry (1993) 16(1-2):247–55. 10.1016/0160-2527(93)90026-b 8500966

[7] Schanda H Ortwein-Swoboda G Knecht G Gruber K The situation of forensic psychiatry in Austria – setback or progress? Int J Law Psychiatry (2000) 5(23):481–92. 10.1016/s0160-2527(00)00045-5 11143946

[8] Salize HJ Dreßing H Placement and treatment of mentally ill offenders–legislation and practice in EU Member States. Final Report. Mannheim: Central Institute of Mental Health (2005).

[9] Buchanan A Grounds A Forensic psychiatry and public protection. Br J Psychiatry (2011) 198(6):420–3. 10.1192/bjp.bp.111.095471 21628701

[10] Gunn J Taylor P Forensic Psychiatry: Clinical, Legal and Ethical Issues. London: Routledge (2014).

[11] Völlm B Bartlett P McDonald R Ethical issues of long-term forensic psychiatric care. Ethics Med Public Health (2016) 2(1):36–44. 10.1016/j.jemep.2016.01.005

[12] Edworthy R Sampson S Völlm B Inpatient forensic-psychiatric care: legal frameworks and service provision in three European countries. Int J Law. Psychiatry (2016) 47:18–27. 10.1016/j.ijlp.2016.02.027 27055603

[13] Völlm BA Clarke M Herrando VT Seppänen AO Gosek P Heitzman J European Psychiatric Association (EPA) guidance on forensic psychiatry: evidence based assessment and treatment of mentally disordered offenders. Eur Psychiatry (2018) 51:58–73. 10.1016/j.eurpsy.2017.12.007 29571072

[14] Hilterman EL Philipse MW de Graaf ND Assessment of offending during leave: development of the Leave Risk Assessment in a sample of Dutch forensic psychiatric patients. Int J Forens. Ment Health (2011) 10(3):233–43. 10.1080/14999013.2011.598601

[15] Walker A Farnworth,L Lapinski S A recovery perspective on community day leaves. J Foren. Pract (2013) 15(2):109–18. 10.1108/14636641311322296

[16] BayMRVG Gesetz über den Vollzug der Maßregeln der Besserung und Sicherung sowie dereinstweiligen Unterbringung (Bayerisches Maßregelvollzugsgesetz – BayMRVG) Vom 17. Juli 2015 (GVBl S. 222) BayRS 312-3-A (Art. 1–54) (2015). Available at: https://www.gesetze-bayern.de/Content/Document/BayMRVG?AspxAutoDetectCookieSupport=1 (Accessed April 8, 2020).

[17] Müller-Isberner R Born P Eusterschulte B Eucker S Praxishandbuch Maßregelvollzug: Grundlagen, Konzepte und Praxis der Kriminaltherapie. Berlin: Medizinisch Wissenschaftliche Verlagsgesellschaft. (2017)

[18] Campbell MA French S Gendreau P The prediction of violence in adult offenders: A meta-analytic comparison of instruments and methods of assessment. Crim. Just. Behav (2009) 36(6):567–90. 10.1177/0093854809333610

[19] Douglas T Pugh J Singh I Savulescu J Fazel S Risk assessment tools in criminal justice and forensic psychiatry: the need for better data. Eur Psychiatry (2017) 42:134–7. 10.1016/j.eurpsy.2016.12.009 PMC5408162 28371726

[20] Hanson RK Morton-Bourgon KE The characteristics of persistent sexual offenders: a meta-analysis of recidivism studies. J Consult. Clin Psychol (2005) 73(6):1154–63. 10.4324/9781351161565-5 16392988

[21] Singh JP Fazel S Gueorguieva R Buchanan A Rates of violence in patients classified as high risk by structured risk assessment instruments. Br J Psychiatry (2014) 204(3):180–7. 10.1192/bjp.bp.113.131938 PMC3939440 24590974

[22] Yang M Wong SC Coid J The efficacy of violence prediction: a meta-analytic comparison of nine risk assessment tools. Psychol Bull (2010) 136(5):740. 10.1037/a0020473 20804235

[23] Ramesh T Igoumenou A Montes MV Fazel S Use of risk assessment instruments to predict violence in forensic psychiatric hospitals: a systematic review and meta-analysis. Eur Psychiatry (2018) 52:47–53. 10.1016/j.eurpsy.2018.02.007 29626758 PMC6020743

[24] Martin K McGeown M Whitehouse M Stanyon W Who’s going to leave? An examination of absconding events by forensic inpatients in a psychiatric hospital. J Forens. Psychiatry Psychol (2018) 29(5):810–23. 10.1080/14789949.2018.1467948

[25] Scott R Goel V Neillie D Stedman T Meehan T Unauthorised absences from leave from an Australian security hospital. Australas. Psychiatry (2014) 22(2):170–3. 10.1177/1039856214522529 24526793

[26] Dickens GL Barlow EM Therapeutic leave from secure mental health inpatient services: a review. In: F. de Jong, M. Liem, J. van Mulbregt (Eds.), Daad, dader en deskundige: liber amicorum prof. dr. Frans Koenraadt. Willem Pompe Instituut, Boomjuridisch. (2018). p. 109–22.

[27] Lyall M Bartlett A Decision making in medium security: Can he have leave? J Forens. Psychiatry Psychol (2010) 21(6):887–901. 10.1080/14789949.2010.500740

[28] Simpson AI Penney SR Fernane S Wilkie T The impact of structured decision making on absconding by forensic psychiatric patients: results from an AB design study. BMC Psychiatry (2015) 15(1):103. 10.1186/s12888-015-0474-1 25935745 PMC4424885

[29] Hart,SD Douglas KS Guy LS The Structured Professional Judgement Approach to violence risk assessment: Origins, nature, and advances. In: Boer DP, editor. The Wiley handbook on the theories, assessment and treatment of sexual offending. Oxford, UK: John Wiley & Sons (2016). p. 643–66.

[30] Etikan I Musa SA Alkassim RS Comparison of convenience sampling and purposive sampling. Am J Theor Appl Stat (2016) 5(1):1–4. 10.11648/j.ajtas.20160501.11

[31] Fakis A Hilliam R Stoneley H Townend M Quantitative analysis of qualitative information from interviews: A systematic literature review. J Mix. Methods Res (2014) 8(2):139–61. 10.1177/1558689813495111

[32] Spencer L Ritchie J O’Connoer W Analysis: practices, principles and processes. In: Ritchie J Lewis J, editors. Qualitative research practice. London: Sage Publications (2004). p. 199–218.

[33] Stufflebeam DL Guidelines for developing evaluation checklists: the checklists development checklist (CDC). Kalamazoo, MI: The Evaluation Center (2000).

[34] Kury H Brandenstein M Riegl M Zur Bedeutung von externen Kriminalprognosen für Vollzugsentscheidungen. In: Strafverteidigung im Rechtsstaat. Baden-Baden: Nomos (2009). p. 976–1002. 10.5771/9783845216942-976

[35] Müller JL Saimeh N Briken P Eucker S Hoffmann K Koller M Standards für die Behandlung im Maßregelvollzug nach §§ 63 und 64 StGB [Standards for treatment in forensic committment according to § 63 and § 64 of the German criminal code: Interdisciplinary task force of the DGPPN]. Nervenarzt (2017) 88(1):1–29. 10.1007/s00115-017-0382-3 28776213

[36] Nedopil N Gross G Prognosen in der Forensischen Psychiatrie: ein Handbuch für die Praxis. Lengerich: Pabst Science Publishers (2005).

[37] Cooper BS Griesel D Yuille JC Clinical-forensic risk assessment: The past and current state of affairs. J Forensic Psychol Pract (2008) 7(4):1–63. 10.1300/J158v07n04_01

[38] Douglas KS Webster CD The HCR-20 violence risk assessment scheme: Concurrent validity in a sample of incarcerated offenders. Crim. Justice Behav (1999) 26(1):3–19. 10.1177/0093854899026001001

[39] Douglas KS Hart SD Webster CD Belfrage H Historical clinical risk management-20 version 3. In: . Assessing risk of violence – User guide. Burnaby, Canada: Mental Health, Law, and Policy. Institute, Simon Fraser University (2013).

[40] Guy LS Packer IK Warnken W Assessing risk of violence using structured professional judgment guidelines. J Forensic Psychol Pract (2012) 12(3):270–83. 10.1080/15228932.2012.674471

[41] Rettenberger M von Franqué F eds. Handbuch kriminalprognostischer Verfahren. Göttingen: Hogrefe (2013).

[42] Riegl M Die Qualität forensischer Prognosegutachten bei Gewalt-und Sexualstraftätern. München: GRIN Verlag (2009).

[43] Webster CD Eaves D Douglas KS Wintrup A The HCR-20 scheme: the assessment of dangerousness and risk–version 1. Burnaby: Simon Fraser University and Forensic Psychiatric Services Commission of British Columbia (1995).

[44] Wertz M Kury H Rettenberger M Umsetzung von Mindestanforderungen für Prognosegutachten in der Praxis. Forensische Psychiatr. Psychol. Kriminol. (2018) 12(1):51–60. 10.1007/s11757-017-0458-8

[45] World Health Organization The international classification of diseases (10th rev.). Geneva: Author (1992).

[46] Andrews D Bonta J The Psychology of Criminal Conduct. (4th ed.) Cincinnati: Anderson (2006).

[47] ZPID (Accessed Accessed September 16, 2019). Available at: https://leibniz-psychology.org/en/institute/about/.

[48] ZPID - Leibniz-Zentrum für Psychologische Information und Dokumentation Verzeichnis Testverfahren. Kurznamen. Langnamen. Autoren. Testrezensionen. 25th edition Trier: ZPID (2018). Available at: https://www.psyndex.de/pub/tests/verz_teil1.pdf.

[49] Singh JP International Perspectives on Forensic Risk Assessment: Measuring Use, Perceived Utility, and Research Quality [Dissertation]. Konstanz: University of Konstanz (2016). Available at: http://nbn-resolving.de/urn:nbn:de:bsz:352-0-322142.

[50] Jick TD Mixing qualitative and quantitative methods: Triangulation in action. Admin. Sci Quart. (1979) 24(4):602–11. 10.2307/2392366

[51] R Core Team R: A language and environment for statistical computing. Vienna, Austria: R Foundation for Statistical Computing (2013). Available at: http://www.R-project.org/.

[52] Chen Y Yang K Marušić A Qaseem A Meerpohl JJ Flottorp S A reporting tool for practice guidelines in health care: the RIGHT statement. Ann Intern Med (2017) 166(2):128–32. 10.7326/m16-1565 27893062

